# Disruption of Gene *pqq*A or *pqq*B Reduces Plant Growth Promotion Activity and Biocontrol of Crown Gall Disease by *Rahnella aquatilis* HX2

**DOI:** 10.1371/journal.pone.0115010

**Published:** 2014-12-11

**Authors:** Lei Li, Ziwei Jiao, Lauren Hale, Wenliang Wu, Yanbin Guo

**Affiliations:** 1 Department of Ecological Science and Engineering, College of Resources and Environmental Sciences, China Agricultural University, Beijing, China; 2 Beijing Key Laboratory of Biodiversity and Organic Farming, China Agricultural University, Beijing, China; 3 College of Chemistry and Biological Sciences, Yi Li Normal University, Yining, Xinjiang, China; 4 Department of Environmental Sciences, University of California Riverside, Riverside, California, United States of America; University of Milan, Italy

## Abstract

*Rahnella aquatilis* strain HX2 has the ability to promote maize growth and suppress sunflower crown gall disease caused by *Agrobacterium vitis, A. tumefaciens,* and *A. rhizogenes*. Pyrroloquinoline quinone (PQQ), a cofactor of aldose and alcohol dehydrogenases, is required for the synthesis of an antibacterial substance, gluconic acid, by HX2. Mutants of HX2 unable to produce PQQ were obtained by in-frame deletion of either the *pqq*A or *pqq*B gene. In this study, we report the independent functions of *pqq*A and *pqq*B genes in relation to PQQ synthesis. Interestingly, both the *pqq*A and *pqq*B mutants of *R. aquatilis* eliminated the ability of strain HX2 to produce antibacterial substance, which in turn, reduced the effectiveness of the strain for biological control of sunflower crown gall disease. The mutation also resulted in decreased mineral phosphate solubilization by HX2, which reduced the efficacy of this strain as a biological fertilizer. These functions were restored by complementation with the wild-type *pqq* gene cluster. Additionally, the phenotypes of HX2 derivatives, including colony morphology, growth dynamic, and pH change of culture medium were impacted to different extents. Our findings suggested that *pqq*A and *pqq*B genes individually play important functions in PQQ biosynthesis and are required for antibacterial activity and phosphorous solubilization. These traits are essential for *R. aquatilis* efficacy as a biological control and plant growth promoting strain. This study enhances our fundamental understanding of the biosynthesis of an environmentally significant cofactor produced by a promising biocontrol and biological fertilizer strain.

## Introduction

The gram-negative bacterium *Rahnella aquatilis* is widely ubiquitous, thriving in soil, water, marshes, and on food, seeds and plant roots. Strains of *R. aquatilis* fix nitrogen in the rhizosphere, solubilize mineral phosphate, and have biocontrol capabilities [1], [2], [3]. Specifically, *R. aquatilis* HX2 has been shown to suppress sunflower crown gall disease caused by *Agrobacterium vitis*, *A. tumefaciens*, and *A. rhizogenes* [4]. Biocontrol activity by *R. aquatilis* is nonspecific. This species has demonstrated suppression of diseases caused by *Xanthomonas campestris*, *X. axonopodis*, *Penicillium expansum*, *Botrytis cinerea,* and *Erwinia amylovora* [5], [6], [7]. Competitive colonization of varied environments and the ability of HX2 to establish several beneficial interactions with plants make it an ideal candidate for a soil inoculant. A mechanistic understanding of plant disease suppression and mineral phosphate solubilization by a *R. aquatilis* was provided in previous research, which linked these activities to the glucose dehydrogense cofactor, pyrroloquinoline quinone (PQQ) [8], [9].

In gram-negative bacteria, PQQ mainly functions as a non-covalently bound, redox cofactor of several membrane-associated sugar and alcohol dehydrogenases, including methanol dehydrogenase, ethanol dehydrogenase, and glucose dehydrogenase (GDH) [10]. Previous reports indicate that the GDH-PQQ holoenzyme is involved in the production of an antimicrobial substance by several genera of bacteria including *Rahnella* and notably, strain HX2 [9], [11], [12]. Organic acids (OA) such as gluconic acid (GA) are considered to be a main factor responsible for dissolution of insoluble phosphate through organometallic complex formation or through metal chelation processes [13], [14]. Bacterial mineral phosphate solubilization (MPS) activity most commonly occurs when bacteria produce and release OAs [14], [15], [16]. Furthermore, PQQ is a plant growth promotion factor, which in addition to GA production, has also been related to its antioxidant properties as well as unknown mechanisms [17].

Bacterial genes involved in PQQ biosynthesis have been identified in numerous species isolated from varying environments and are clustered in *pqq*ABCDEF operons [9], [18], [19], [20]. The *pqq*A gene encodes a small peptide that contains tyrosine and glutamate and serves as the precursor and rate-determining step for PQQ biosynthesis [21]. This molecule remains attached to a precursor peptide and is cleaved off at a later step by other enzymes of the biosynthesis pathway. Often, *pqq*B is not directly required for PQQ biosynthesis. Its suggested role in *K. pneumoniae* is a carrier that facilitates the secretion of PQQ across the plasma-membrane into the periplasm [22]. Little information is available on PQQ biosynthesis in the *Rahnella* genus. Kim et al. [8] mobilized a cosmid library of *R. aquatilis* into *Escherichia coli* HB101 to isolate and clone the genes that confer the MPS trait from *R. aquatilis*. Consequently, it was revealed that the MPS locus of *R. aquatilis* contains the *pqq*D and *pqq*E genes. Mutants of *R. aquatilis* HX2 showed that a lack of antibacterial activity was due to a Tn5 insertion in the *pqq*E gene, which prevented synthesis of the PQQ [9]. The aim of our study is to investigate the individual roles of *pqq*A and *pqq*B genes in *R. aquatilis* HX2 PQQ biosynthesis. The relevance of these genes to growth and beneficial activities of HX2 are assessed with respect to synthesis of OAs, antibacterial activity, mineral phosphate solubilization, biological control of crown gall disease, and plant growth promotion.

## Materials and Methods

### Bacterial Strains, Plasmids, and Culture Conditions

The bacterial strains, plasmids, and primers used in this study are listed in Table 1. *R. aquatilis* strains were cultured at 28°C on potato dextrose agar (PDA) medium or with shaking (170 rpm) in potato dextrose broth (PDB) [3], [9]. The *E. coli* strains DH5α [23] and DH5α (λ-pir) were grown at 37°C on Luria-Bertani (LB) medium. To test PQQ production, cultures were grown in AB minimal medium (containing, per liter: K_2_HPO_4_, 3 g; NaH_2_PO_4_, 1 g; NH_4_C1, 1 g; MgSO_4_.7H_2_O, 0.3 g; KCl, 0.15 g; CaCl_2_, 0.01 g; FeSO_4_.7H_2_O, 2.5 mg; glucose, 0.5%) [24]. *Agrobacterium vitis* strain K308 [25] was grown either on yeast extract broth (YEB) or yeast extract agar (YEA) at 28°C [26]. When required, media supporting the growth of *R. aqautilis* and *E. coli* were supplemented with filter-sterilized antibiotics (kanamycin, 50 µg ml^−1^; ampicillin, 50 µg ml^−1^), isopropyl-β-D-thiogalactopyranoside (IPTG) at 1 mM, and 5-bromo-4-chloro-3-indolyl-β-D-galactopyranoside (X-Gal) at 40 µg ml^−1^. All of the studies involving *R. aquatilis* HX2 inoculation were carried out in a closed and protected greenhouse at the China Agricultural University. This study did not involve endangered or protected species.

**Table 1:** Bacterial strains, plasmids^a^ and primers.

Strains/Plasmids/Primers	Characteristics	Source or reference
Strains		
***Escherichia coli***		
DH5α	F^−^ *recA1 endA1 hsdR17 supE44 thi-1 gyrA96 relA1*Δ (*argE-lacZYA*)*169*Ф*80lazA*Δ*M15*	[23]
DH5α (λpir)	F^−^ *recA1 endA1 hsdR17 supE44 thi-1 gyrA96 relA1*Δ (*argE-lacZYA*)*169*Ф*80lazA*Δ*M15 λpir*	[23]
***Rahnella aquatilis***		
HX2	Ap^R^, Wild type,ABS^+^, biocontrol	[3]
HX2▵A	Ap^R^, HX2 derivative with a 32 bp deletion in *pqqA* gene, ABS^-^,reduced biocontrol	This study
HX2▵B	Ap^R^, HX2 derivative with a 564 bp deletion in *pqqB* gene, ABS^-^, reduced biocontrol	This study
CHX2▵A	Ap^R^, Tc^R^, HX2▵A containing plasmid pCH15 with the *pqq* genes, complemented strain, ABS^+^,biocontrol	This study
CHX2▵B	Ap^R^, Tc^R^, HX2▵B containing plasmid pCH15 with the *pqq* genes, complemented strain, ABS^+^,biocontrol	This study
***Agrobacterium vitis***		
K308	Pathogen of grapevine crown gall, octopine type Ti plasmid	[25]
**Plasmids**		
pBluescript II SK+	Ap^R^, ColE 1 origin, Cloning vector	Stratagene (La Jolla, CA)
pBSNot6	Ap^R^, a NotI site inserted into pBluescript following the KpnI site	[30]
pMD18-T	Ap^R^, ColE 1origin, T-vector	TaKaRa Bio (Kyoto, Japan)
pSR47S	Km^R^, R6K*oriV* RP4*oriT sacB*	[31]
pRK415G	Gm^R^, Tc^R^, Broad-host-rang cloning vector, IncP1 replicon; polylinker of pUC19	[33]
pRK600	Cm^R^, ColE1 *oriV*; RP4; *tra* ^+^; RP4 *oriT*; helper plasmid in triparental matings	[32]
pBSNot6▵pqqA	Ap^R^, pBSNot6 containing a 2685 bp *Sal* I-*Eco*R I fragment with *pqqA* gene deletion	This study
pBSNot6▵pqqB	Ap^R^, pBSNot6 containing a 1940 bp *Eco*R I-*Sal* I fragment with *pqqB* gene deletion	This study
pSR47S▵pqqA	Km^R^, pSR47S containing 2685 bp fragment with a 32 bp deletion in *pqqA*	This study
pSR47S▵pqqB	Km^R^, pSR47S containing 1940 bp fragment with a 564 bp deletion in *pqqB*	This study
** Primers**		
ΔpqqA-L1(*Sal* I)	5′-TAGTCGACTGCTGCCCTGTTTCTTG-3′	This study
ΔpqqA-L2(*Bam*H I)	5′-ATGGATCCACATAATTACGTCCTCTTG-3′	This study
ΔpqqA-R1(*Bam*H I)	5′-ATGGATCCTGCTTAGAAGTGACGCTGTAC-3′	This study
ΔpqqA-R2(*Eco*R I)	5′-ATGAATTCAGTAACCGTAACCTTCTTCCTC-3′	This study
ΔpqqB-L1(*Eco*R I)	5′-ATGAATTCTGGATTGCTGCGTGAGTGT-3′	This study
ΔpqqB-L2(*Bam*H I)	5′-TAGGATCCTTCAATCTGGTGGCAAATGTC-3′	This study
ΔpqqB-R1(*Bam*H I)	5′-ATGGATCCTGATTGCCCTGCTGTCT-3′	This study
ΔpqqB-R2(*Sal* I)	5′-ATGTCGACGTGTAGTGTTCGGTAATGG-3′	This study

a Ap^R^, Cm^R^, Km^R^, Gm^R^ and Tc^R^ indicate resistance to ampicillin, chloromycetin, kanamycin, gentamicin and tetracycline, respectively.

b ABS^+^ and ABS^−^ indicate production antibacterial substance or not.

c Underlined bases are restriction enzyme cut sites.

### General Genetic Techniques

Isolation of genomic DNA from strain HX2 and plasmid DNA from *E. coli* were performed according to standard procedures [27]. Restriction enzyme digestions were performed as recommended by the suppliers (TaKaRa, Japan) and ligations were carried out using T4 DNA ligase (TaKaRa, Japan). Gel electrophoresis was performed in 0.8–1.0% agarose gels. For cloning purposes, *Ex™ Taq* DNA polymerase (TaKaRa, Japan) was used to PCR amplify inserts and *Taq* DNA polymerase (TaKaRa, Japan) was used for PCR amplification in test reactions (e.g., colony PCR). DNA sequencing was performed by Invitrogen Life Technologies (Beijing, China) and analyzed by using the National Center for Biotechnology Information BLAST server (http://www.ncbi.nlm.nih.gov/BLAST). The partial genome sequence of *R. aquatilis* HX2 (accession number CP003403-6) was used for primer design [28]. Primers used in this study are listed in Table 1.

### Construction of *pqq*A and *pqq*B In-frame Deletion Mutants

In-frame nonpolar deletions of *pqq*A and *pqq*B were constructed utilizing a two-step homologous recombination strategy as described previously [29]. Primers were designed based on sequences upstream and downstream of either *pqq*A or *pqq*B and were used to PCR amplify fragments from the genome of HX2 (Table 1). Briefly, primers ΔpqqA-L1 and ΔpqqA-L2 with sites for *Sal* I and *Bam*H I, were used to amplify a 1132 bp region upstream of the *pqq*A open reading frame (ORF). A 1553 bp fragment, created by primers ΔpqqA-R1 and ΔpqqA-R2 containing *Bam*H I and *Eco*R I sites, is downstream of the *pqq*A ORF (Table 1). The standard PCR reactions involved 15 min at 94°C, then 35 cycles of 45 s at 94°C, 40 s at 66°C and 1 min at 72°C, and final extension at 72°C for 10 min. Similarly fragments flanking *pqq*B were amplified. These included a 982 bp upstream fragment that included the first 193 codons of the *pqq*B ORF, generated from primers ΔpqqB-L1 and ΔpqqB-L2 with sites *Eco*R I and *Bam*H I and a 958 bp downstream fragment including the last 171 codons of the *pqq*B ORF from primers ΔpqqB-R1 and ΔpqqB-R2 with *Bam*H I and *Sal* I sties. These PCR reactions were carried out at 94°C for 15 min, followed by 35 cycles of 94°C for 45 s, 71°C for 2 min, and a final extension at 72°C for 10 min. After being digested with appropriate restriction enzymes, the ΔpqqA-L/R and ΔpqqB-L/R fragments were ligated into pBSNot6 [30] creating pBSNot6ΔpqqA and pBSNot6ΔpqqB. Then, the approximately 2.7 kb Not I fragment from pBSNot6ΔpqqA and the approximately 2.0 kb Not I fragment from pBSNot6ΔpqqB, including a *pqq*A gene with 32 bp deletion and a *pqq*B gene with 564 bp deletion, were lifted and ligated into pSR47S [31] to obtain pSR47SΔpqqA and pSR47SΔpqqB. The two suicide plasmids were transformed into *E. coli* DH5α (λ-pir) by heat shock and were mobilized from DH5α (λ-pir) into the wild-type *R. aquatilis* strain HX2 by triparental mating with helper strain DH5α carrying plasmid pRK600 [32]. Exconjugants were selected on AB minimal agar plates containing kanamycin and second recombination events were selected according to the methods previous described [9]. The mutants of HX2ΔA and HX2ΔB were each screened from approximately 1500 first recombination clones for the absence of kanamycin resistance. A fragment 33 bp from the *pqq*A 72 bp gene was deleted in mutant HX2ΔA and 564 bp of the 912 bp *pqq*B gene was deleted in HX2ΔB. The disruption of each gene and the absence of the vector within the genome of these mutants were confirmed by PCR with primers ΔpqqA-L1/ΔpqqA-R2 and ΔpqqB-L1/ΔpqqB-R2 and sequencing analysis.

### Genetic Complementation of the *pqq*A and *pqq*B Mutants

To complement the *pqq* mutants an 8.0 kb *Bam*H I fragment containing the entire *pqq*ABCDEF operon of HX2 was cloned into the broad host range vector pRK415G [33], resulting in the complementation plasmid pCH15 [9]. The plasmid pCH15 was mobilized into the HX2▵A or HX2▵B strain by triparental mating, and the complement strains CHX2▵A and CHX2▵B were created.

### Antibiosis Test in Vitro and Biocontrol of Crown Gall Disease in Greenhouse

The antagonist HX2 and its derivative strains were tested for in vitro antibiosis against pathogenic strain *A. vitis* K308 via a modified Stonier's method described by Chen et al [3]. Biocontrol activity assays were performed on sunflower (*Helianthus annuus* L.) stems with two true leaves grown in a greenhouse according to a previously described method [3]. Briefly, a suspension of pathogenic agrobacterial strain K308 (ca. 2×10^8^ CFU ml^−1^) was mixed with an equal volume of HX2 or its derivative strains suspension (ca. 2×10^8^ CFU ml^−1^). A 10 µl drop of this mixture was injected into a 1.0 cm longitudinal incision in sunflower stem. The inoculation site was wrapped with Parafilm. Gall formation was observed, and the gall was excised and weighed 15 days after inoculation. Sterile buffered saline (SBS, 0.85% NaCl) was applied as a negative control and K308 mixed with SBS served as a positive control. The effectiveness index (EI) was calculated using the following formula: EI (%)  =  [(C-T)/C]×100, where C is the mean fresh weight of the crown gall tumor of the positive control group and T is the mean fresh weight of the crown gall tumor in the treated group. The assay was performed with 4 replicates and 10 plants were used per treatment.

### Determination of Mineral Phosphate Solubilization and pH

HX2 and derivative strains were inoculated and shaken in PDB at 28°C, 170 rpm for 48 h. Ten microliters of bacteria culture were dropped on a sterile filter paper (diameter, 5 mm), placed in the middle of agar plates containing the differential medium, national botanical research institute's phosphate (NBRIP). The NBRIP growth medium contains (per liter): glucose, 10 g; Ca_3_(PO_4_)_2_, 5 g; MgCl_2_.6H_2_O, 5 g; MgSO_4_.7H_2_O, 0.25 g; KCl, 0.2 g; (NH_4_)_2_SO_4_, 0.1g [34] with 18 g agar. The phosphate-solubilizing halo diameter was measured after the plates were incubated at 28°C for 7 days.

HX2 and derivative strains were cultured in liquid NBRIP at 28°C for 7 days to detect the soluble phosphorus (P) in the medium. Two-milliliters of bacterial supernatants were collected by centrifuge at 12,000 g for 5 min every other day. Soluble phosphate in the culture supernatants was detected using the Molybdenum-blue method [35]. Simultaneously, pH of the corresponding NBRIP medium was detected using a pH monitor (Mettler Toledo FE20, Shanghai, China). This assay was performed in triplicate with 3 replicates per treatment. Results from the third experiment are reported here.

### Greenhouse Experiments for Plant Growth Promotion

Soil and coarse sand (0.35–0.5 mm) (1∶1, w/w) were air-dried, passed through a sieve (2 mm), and sterilized by autoclaving at 121°C for 2 h before filling the pots. The soil texture is sandy loam (70.8% sand, 26.9% silt, and 2.3% clay). The soil type is a calcaric cambisol according to the FAO/UNESCO soil map of the world. Maize caryopses (*Zea may* L., Zhengdan 958, Henan Academy of Agricultural Sciences, China) were surface sterilized with 70% ethanol for 30 sec, washed with sterilized distilled water for three times, and germinated in a sterilized Petri dish (270 mm) for 2 days. The bacteria strains were cultured with PDB at 28°C for 2 days and diluted to OD_600_ of 0.6 (ca. 2×10^8^ CFU ml^−1^). Maize seeds were coated with the diluted bacteria suspension or control (PDB) for 3 h before sowing. Coated maize caryopses were cultivated individually in pots (diameter 180 mm, length 160 mm) containing 750 g of soil (P_2_O_5_, 6.53 mg kg^−1^; soil organic matter, 18.74 g kg^−1^; pH, 7.49) amended with rock phosphate (1%, w/w), and 20 ml Hoagland's nutrition liquid without phosphorus (containing per liter) [Ca(NO_3_)_2_, 945 mg; KNO_3_, 607 mg; MgSO_4_, 493 mg and 2.5 ml Ferrum salt solution (per liter) FeSO_4_ 7H_2_O, 5.56 g; EDTA, 7.46 g, pH 5.5)]. The pots were maintained in a greenhouse and irrigated with 50 ml sterile water every other day. After 42 days the plants were harvested and lengths and fresh weights of the plants were determined. The dry weights of shoots and roots were measured following drying at 65°C for 48 hours. Total P of plants and soluble P of soils were detected according to the methods of Murphy and Riley [35]. The experiments were repeated 3 times and 20 plants were used in each treatment.

### Detection of PQQ

The presence of PQQ in culture supernatants was determined as described previously [9]. In brief, bacterial strains were grown at 28°C in AB minimal medium for 48 h, cell cultures were then mixed with methanol at a 1∶9 ratio (v/v). Precipitated material was removed by centrifugation (12,000 g, 15 min) and the methanol was evaporated with rotary evaporator. The sample was acidified with HCl to pH 2.0 and loaded onto a Sep-Pak C_18_ cartridge (Agilent Technologies, USA). The cartridge was washed with 20 ml of 2 mM HCl and then PQQ was eluted with 70% methanol. To identify the PQQ peak, 200 µl of the sample was mixed with 100 µl of 0.2 M Na_2_B_4_O_7_ buffer, adjusted to pH 8.0 with HCl, and mixed with 90 µl 0.5% acetone, then incubated for 30 min at room temperature. Reverse-phase high-performance liquid chromatography (RP-HPLC) was carried out using a Shimadzu LC-6A HPLC system with a fluorescence detector on ZORBAX SB-C_18_ columns (4.6×250 mm, 5 µm; Agilent Technologies, USA) eluted with 27% methanol and 0.4% H_3_PO_4_ at a flow rate of 0.8 ml min^−1^. The excitation and detection wavelengths of the fluorescence detector were set at 360 nm and 480 nm.

### Identification of Organic Acid

Organic acids (OA) produced by bacterial strains were detected as described previously with some modifications [36]. HX2 and derivative strains were cultivated in NBRIP medium at 28°C for 7 days. The culture was centrifuged at 12,000 g for 5 min and filtrated through a 0.22 µm filter (Pall Corporation, USA). Twenty microliters of filtrates were injected to high-performance liquid chromatography (HPLC) (Waters 2998, USA) equipped with ZORBAX SB-C_18_ columns (4.6×250 mm, 5 µm; Agilent Technologies, USA). The chromatogram class (NH_4_)_2_HPO_4_ (0.5%, w/v) with a pH of 2.81 was used as mobile phase at flow rate of 0.4 ml min^−1^. UV absorption was routinely monitored at a wavelength of 214 nm. Five types of OA served as standards for all samples; gluconic acid (GA), lactic acid (LA), citric acid (CA), succinic acid (SA) and propionic acid (PA).

### Statistical analysis

In this study, mean values among treatments were compared by Duncan's Multiple Range test at Ρ<0.05. Analysis of variance (ANOVA) was performed on the data using SAS software (version 8.2; SAS, Inc., Cary, NC).

## Results

### Antibiotic Production and Biocontrol are Associated with *pqq*A and *pqq*B

**Figure 1:**
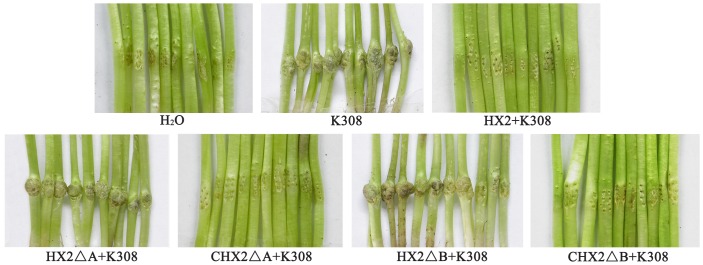
Biological control crown gall disease of sunflower with *R. aquatilis* HX2 and derivative strains. Crown gall on sunflower were caused by *A. vitis* K308 with wild-type HX2 and derivative strains including *pqq*A mutant strain HX2ΔA, *pqq*B mutant strain HX2ΔB, complemented mutants CHX2ΔA and CHX2ΔB whilst water as control.

The *pqq*A and *pqq*B in-frame deletion mutants HX2ΔA and HX2ΔB lacked the ability to inhibit growth of *A. vitis* K308 on PDA (Table 2). Strains CHX2ΔA and CHX2ΔB, which were HX2ΔA and HX2ΔB strains complemented by plasmids containing the *pqq* gene cluster, displayed phenotypes of *A. vitis* K308 inhibition *in vitro* (Table 2). Strain HX2ΔA and HX2ΔB showed lesser biocontrol activity to crown gall disease on sunflower than wild-type strain HX2 and complement strain CHX2ΔA and CHX2ΔB (Table 2). Strain HX2ΔB showed higher biocontrol efficiency compared to strain HX2ΔA and EI of crown gall disease treated by HX2ΔB significantly increased by 41% compared to HX2ΔA treatment (P<0.05) (Table 2 and Fig. 1). The abilities to produce antibacterial substance, inhibit growth of *A. vitis* K308 *in vitro*, and suppress gall development on sunflower were fully restored in the complemented strain CHX2ΔA and CHX2ΔB, respectively (Table 2 and Fig. 1).

**Table 2:** Production of PQQ and inhibition effect of *Rahnella aquatilis* HX2 and its derivatives on the growth of *Agrobacterim vitis* strain K308 and tumor formation on sunflowers.

Strains	PQQ (ng/ml)	Inhibition zone diameter (mm)	EI (%)
HX2	8.21±0.02 a	30.3±0.3 a	89.4±2.9 a
HX2▵A	\	\	19.5±1.1 c
HX2▵B	\	\	27.4±1.0 b
CHX2▵A	8.02±0.02 b	27.3±0.2 a	88.6±1.3 a
CHX2▵B	7.93±0.03 b	28.7±0.3 a	88.9±1.8 a

Mean ± standard error values followed by different letters indicate statistically significant differences (*P*<0.05). The effectiveness index (EI) was calculated using the following formula: EI (%)  =  [(C-T)/C]×100, where C is the mean fresh weight of the crown gall tumor of the positive control group and T is the mean fresh weight of the crown gall tumor in the treated group.

### Genes *pqq*A and *pqq*B are Related to Phosphate Solubilization and Plant Growth Promotion

Based on the growth conditions of the bacteria strains in NBRIP with agar after 7-day incubations, it was shown that there was a significant decrease (P<0.05) of phosphate-solubilizing halo diameters when either the *pqq*A or *pqq*B gene was disrupted (Table 3). When HX2ΔA or HX2ΔB was complemented with the *pqq* gene cluster (CHX2ΔA or CHX2ΔB), phosphate solubilization on NBRIP plates was restored. Wild-type HX2 excreted high levels of OA, especially GA and manifested much stronger MPS ability compared to strains HX2ΔB and HX2ΔA (Table 2). Strains CHX2ΔA and CHX2ΔB restored intrinsic MPS ability and secreted similar levels of GA compared to the wild-type strain HX2. The phosphate-solubilizing halo diameters of *R.aquatilis* HX2, CHX2ΔA, and CHX2ΔB colonies were about two-fold greater than that of HX2ΔA and HX2ΔB strains (Table 3). The phosphate-solubilizing halo of complemented strains CHX2ΔA and CHX2ΔB were, in-fact, even greater than those of the wild type strain HX2 (significant at P<0.05) (Table 3).

**Table 3:** Diameter of phosphate-solubilizing zone, pH and soluble P observed after 7 d incubation in NBRIP.

Strains	Phosphate solubilizing halo diameter (mm)	Soluble P (mg l^−1^)	pH of medium	Gluconic acid (g l^−1^)
HX2	22.7±0.3 b	438.7±15.0 a	3.45±0.01 c	9.68±0.34 ab
HX2▵A	12.7±0.2 c	99.7±3.7 b	4.68±0.03 a	0.65±0.01 d
HX2▵B	13.1±0.1 c	115.3±6.3 b	4.53±0.06 b	2.01±0.46 c
CHX2▵A	24.7±0.3 a	450.3±7.0 a	3.45±0.06 c	8.75±0.19 b
CHX2▵B	25.0±0.6 a	465.9±21.8 a	3.38±0.02 c	10.84±0.68 a

Mean ± standard error values followed by different letters indicate statistically significant differences (*P*<0.05).

To confirm that *pqq*A and *pqq*B were related to phosphate solubilization, additional tests were performed to quantify soluble P and lower culture pH in liquid media (Table 3 and S1 Figure). After 7-day incubations, the concentration of soluble P in culture solutions treated with CHX2ΔA, CHX2ΔB were 450.3 mg l^−1^ and 465.9 mg l^−1^, which were 3.7 and 3.5 times greater than mutant HX2ΔA and HX2ΔB (Table 3). There was significant decrease (P<0.05) in soluble P concentration between strains with a disrupted *pqq*A or *pqq*B gene compared to the wild type strain HX2. At the same time, the pH value of culture solutions increased from 3.45 to 4.68 and 4.53 when the *pqq*A or *pqq*B gene was disrupted (Table 3). Disruption of *pqq*A resulted in the lowest concentration of soluble P (99.7 mg l^−1^) of the derivative strains tested and also had the highest culture pH (4.68). The largest quantity of soluble P (465.9 mg l^−1^) was present in the CHX2ΔB culture, corresponding to the lowest pH (3.38). There was a significant negative correlation (R^2^ = −0.97, P<0.01) between pH and soluble P.

The effects of strains HX2ΔA and HX2ΔB on plant growth promotion were monitored using pot experiments in the greenhouse. The details of the plant length, fresh and dry weight, total P and soluble P at 42 days post-inoculations are listed in Table 4. All plant growth parameters and conditions measured here were improved in the presence of HX2 as compared to the negative control. Specifically, maize plant length increased by 48%, fresh weight was 255% greater, dry weigh increased by 168%, plant total P was 67% greater, and there was 182% more soluble P in the soil. The effects of HX2 and derivative strains on plant growth promotion are significant, as compared to a blank control. However, HX2, CHX2ΔA, and CHX2ΔB treatments were more effective in enhancing maize length, fresh weight, dry weight, total P and soluble P in soil than HX2ΔA or HX2ΔB mutant treatments. There was no significant difference in length or fresh weight among HX2ΔA and HX2ΔB mutant treatments. However, there was a significant difference between HX2ΔB treatment and HX2ΔA treatments in which HX2ΔB displayed 19% greater dry plant weight, 17% more total P in maize, and 18% more soluble P in soil than HX2ΔA. In contrast to the negative control, the effect of HX2ΔB also shows significant improvement with respect to plant dry weight and soil soluble P where as HX2ΔA treatment only resulted in significantly greater soil soluble P. There was a significant positive correlation (R^2^ = 0.87, P<0.01)between fresh weight and soil soluble P. Soil soluble P and NBRIP media soluble P treated by the strains was positively correlated as well (R^2^ = 0.72, P <0.01).

**Table 4:** Green house pot experiment: effect of HX2 and derivative strains on maize plant height and weight and soil total P and soluble P.

Strains	Length (cm)	Fresh weight (g plant^−1^)	Dry weight (g plant^−1^)	Total P (mg kg^−1^)	Soluble P (mg kg^−1^)
CK^a^	54.2±0.9 c	8.12±1.61 c	1.68±0.07 e	1.28±0.05 b	5.12±0.30 e
HX2	80.0±1.9 a	28.82±2.57 a	4.51±0.18 a	2.09±0.01 a	14.46±0.20 a
HX2▵A	70.0±1.1 b	16.24±1.03 b	1.87±0.08 e	1.00±0.04 c	8.09±0.06d
HX2▵B	70.6±0.6 b	16.42±0.99 b	2.22±0.13d	1.17±0.06 b	9.54±0.06 c
CHX2▵A	78.0±2.4 a	25.73±0.51 a	3.52±0.20 c	2.09±0.01 a	11.86±0.10 b
CHX2▵B	76.2±1.0 a	23.94±2.09 a	3.84±0.16 b	2.08±0.01 a	11.79±0.12 b

Mean ± standard error values followed by different letters indicate statistically significant (*P*<0.05).

a CK is negative control, LB medium only.

### Detection of PQQ

**Figure 2:**
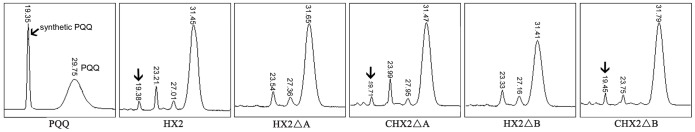
RP-HPLC detection of PQQ synthesized by *R. aquatilis* HX2 and derivative strains. Arrows indicate 5-acetonyl-PQQ.

The level of PQQ production by HX2ΔA and HX2ΔB, determined with HPLC showed that *pqq*A and *pqq*B mutants lost the ability to biosynthesize PQQ (Table 2 and Fig. 2). Non-detectable levels of PQQ were shown for each *pqq* mutant (HX2ΔA and HX2ΔB), but an average of 8.0 ng ml^−1^and 7.9 ng ml^−1^ were detected in complemented strains CHX2ΔA and CHX2ΔB. However, PQQ produced by the complemented strains was significantly less than wild strain HX2 (Table 2).

### OA Production

**Figure 3:**
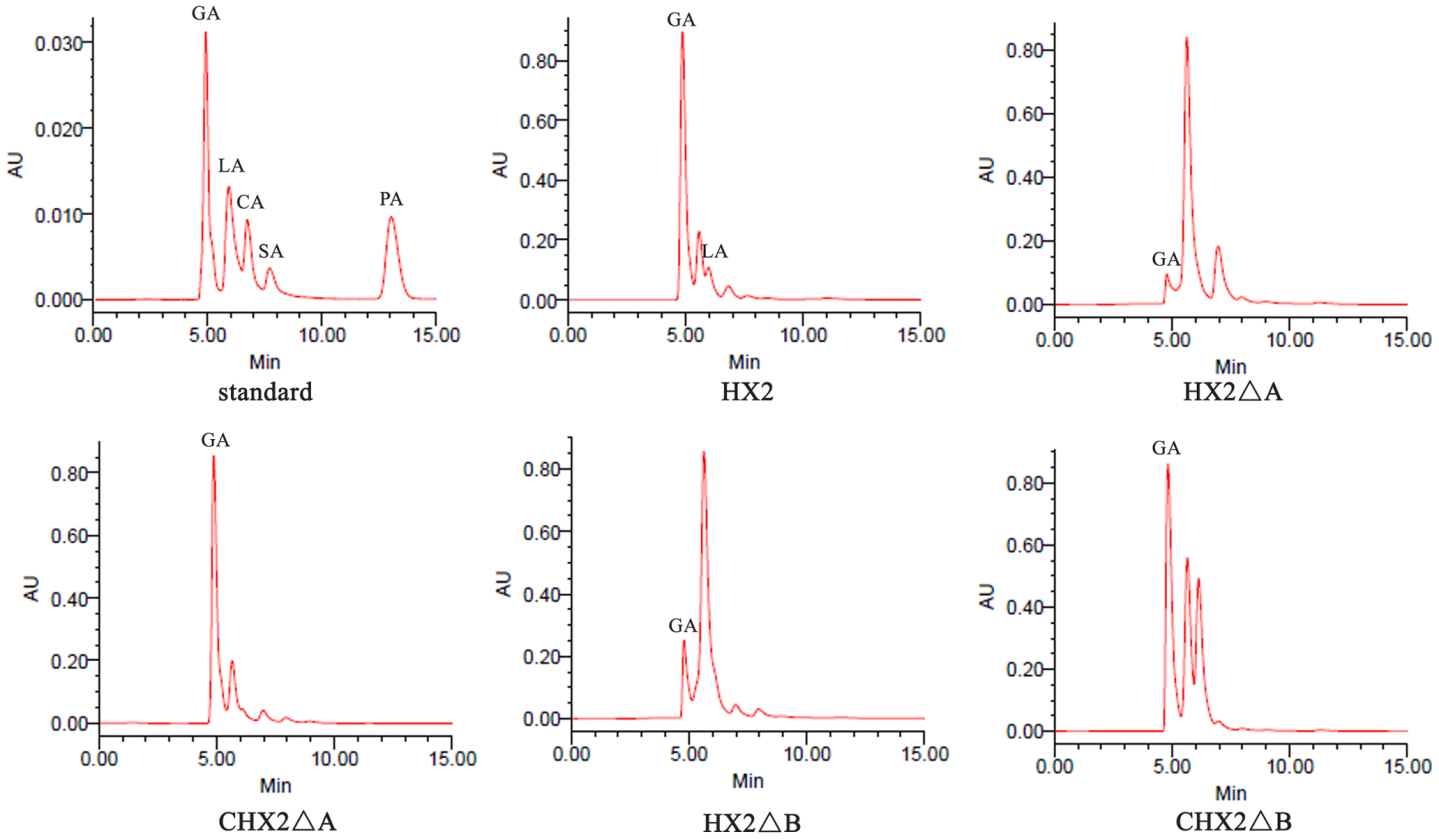
Identification of OAs produced by *R. aquatilis* HX2 and derivative strains.

Gluconic acid was the main OA produced by *R. aquatilis* HX2 which accounted for 94.3% of total OA production (Fig. 3). While HX2 produced GA and LA, HX2ΔA and HX2ΔB with their complemented strains only produced GA (Fig. 3). Strains HX2ΔA and HX2ΔB excreted low quantities of gluconic acid, as compared to all strains with intact *pqq* gene clusters (Table 3). HX2ΔB produced significantly more GA than did HX2ΔA (P<0.05). Furthermore, CHX2ΔB GA production was significantly greater than that of CHX2ΔA (P<0.05). Production of GA shows a significant negative correlation (r = −0.984, P<0.01) to pH of culture solution and is positively correlated (r = 0.973, P<0.01) to amount of soluble P in culture solutions.

## Discussion

The most significant findings of this study are the different outcomes of *pqq*A and *pqq*B mutations on biocontrol capabilities, P solubilization, and plant-growth promotion by strain HX2. Although the *pqq*A gene product is redundant for the synthesis of PQQ in some bacteria, its availability was essential for PQQ biosynthesis in HX2 (Table 2) [37]. Of the derivative strains, HX2ΔA exhibited the lowest amounts of soluble P and excretion of organic acid and cultures had the highest pH. Notably, the HX2ΔB mutant displayed significantly greater GA production, soil soluble P and dry plant weight when compared to HX2ΔA (Table 3 and Table 4). This indicates a different role of *pqq*B in PQQ biosynthesis than that of *pqq*A. Furthermore, when HX2ΔA and HX2ΔB were complemented with the entire *pqq*ABCDEF operon, the derivatives have additional *pqq* genes as compared to the wild-type strain. Forexample, CHX2ΔB now has one *pqq*B gene and duplicate *pqq*A genes. There are several differences between CHX2ΔA and CHX2ΔB. Specifically, CHX2ΔB produces significantly more GA than CHX2ΔA and the wild-type strain. However PQQ was not measured at higher levels for CHX2ΔB. All together, these findings indicate that *pqq*A is more important than *pqq*B in biocontrol capabilities, P solubilization, and plant-growth promotion by strain HX2. These results fall in line with those previously reported. Velterop et al. [22] found that the PqqA protein provided precursors for PQQ biosynthesis and that PqqB proteins facilitated the transport of PQQ across the cytoplasmic membrane into the periplasm in *Klebsiella pneumonia*. In this case PQQ biosynthesis was rate dependent on PqqA [22]. Hence, in HX2 the function of the *pqq*A g and *pqq*B genes may be similar to that in *K. pneumonia*.

Many researchers have shown that the abilities of gram-negative bacteria, such as *R. aquatilis*, *Pseudomonas cepacia,* and *Enterobacter intermedium*, to solubilize insoluble phosphates were dependent upon *pqq* genes [38], [39], [40], [41]. PQQ is necessary for the assembly of the GDH holoenzyme, which acts in the oxidation of glucose to GA [42], [43]. The results described here indicate that the MPS ability of *R. aquatilis* HX2 was mainly determined by GA and LA production, and GA was significantly more important (Table 3 and Fig. 2). The correlation between quantities of GA and soluble P concentration indicates that GA production by HX2 effectively reduces the medium pH to increase MPS ability (Table 3, Fig 2 and S1 Figure). The main mechanism of insoluble phosphate dissolution in strain HX2 relates to glucose metabolism, in which GA is produced under the action of GDH utilizing PQQ as cofactor. Also, GA production will be greater if PQQ biosynthesis is increased. In this strain, *pqq*A and *pqq*B are both required for PQQ biosynthesis and to have wild-type levels of GA production. Although, it was at a greatly reduced level, strain HX2ΔA still produced GA but PQQ production was non-detectable. This brings to question if there is a GDH-PQQ independent pathway for GA catabolism in HX2.

Plant growth promotion was directly related to strain P solubilization ability in soil and culture medium (Table 3 and Table 4). Maize growth promotion was greatest when treatments included HX2, CHX2ΔA, and CHX2ΔB, while HX2ΔA and HX2ΔB treatments demonstrated promotion to a significantly lesser extent. Likewise, strains HX2, CHX2ΔA and CHX2ΔB produced the highest amounts of GA, which released insoluble P into the soil solution (Table 4). This is consistent with previous findings where inoculation of phosphate solubilizing bacteria such as *Serratia marcescens*, *Pseudomonas fluorescens* and *Bacillus* spp. improved the phosphorous uptake of shoots and grains in maize and peanut plants [44], [45], [46]. Even with the greatly reduced GA production levels, HX2ΔA and HX2ΔB had benefits with respect to soil P-solubilization as compared to negative controls. It has been reported that plant growth promotion can be achieved by direct and indirect interaction between beneficial microbes and their host plants [47]. Given that HX2ΔA and HX2ΔB promoted plant growth, it is likely that strain HX2 has other plant-growth promoting properties in addition to phosphate solubilization.

Derivative strain HX2ΔB showed more effective biocontrol than did HX2ΔA (Table 2). This is likely related to greater GA production by HX2ΔB. Gluconic acid can serve as an antifungal agent and has been associated with the regulation of other antimicrobial compounds, 2,4-diacethylphloroglucinol and pyoluteorin [11], [48]. Without PQQ production, HX2ΔA and HX2ΔB mutants had greatly reduced abilities to produce GA and disrupted the ability to produce antibacterial substance, but they were still able to suppress tumor formation on sunflowers significantly (Fig. 1). Hence, it is likely that production antibacterial substance and GA is not the sole mechanism involved in HX2 biocontrol of crown gall disease.

Further work should be done to identify the potential additional plant growth promoting factors and antibacterial substances produced by HX2. More information will provide insight to optimized growing conditions or modifications to the strain which can lend it improved benefits to plants.

## Supporting Information

S1 Figure **Soluble P (a) and pH (b) in media of HX2 and derivative strain cultures.**
(TIF)
