# Supplementary figures and images for: Disruption of Gene pqqA or pqqB Reduces Plant Growth Promotion Activity and Biocontrol of Crown Gall Disease by Rahnella aquatilis HX2
Source: PLoS One. 2014 Dec 11;9(12):e115010. doi: 10.1371/journal.pone.0115010 (PMC4263746; doi:10.1371/journal.pone.0115010)

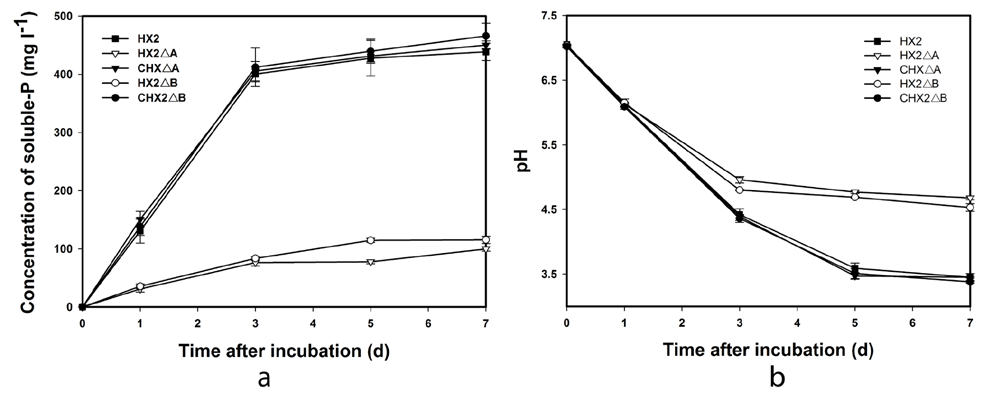

Supplement: S1 Figure — Soluble P (a) and pH (b) in media of HX2 and derivative strain cultures. (TIF) [file pone.0115010.s001.tif]
